# Study on Strengthening Mechanism of 9Cr-1.5Mo-1Co and 9Cr-3W-3Co Heat Resistant Steels

**DOI:** 10.3390/ma13194340

**Published:** 2020-09-29

**Authors:** Long Zhao, Xiangru Chen, Tieming Wu, Qijie Zhai

**Affiliations:** 1 Center for Advanced Solidification Technology (CAST), School of Materials Science and Engineering, Shanghai University, Shanghai 200444, China; zhaolongXYZ@shu.edu.cn (L.Z.); chenxr@shu.edu.cn (X.C.); 2 Shanghai Electric Group Company Limited, Shanghai 200336, China; wutm2@shanghai-electric.com

**Keywords:** heat-resistant steel, cast steel, microalloying, strengthening mechanism

## Abstract

The strengthening mechanism of 9Cr–1.5Mo–1Co and 9Cr–3W–3Co heat resistant steel was studied by tensile test and microstructure analysis. At the same temperature, the yield strength of 9Cr–3W–3Co heat-resistant steel is higher than that of 9Cr–1.5Mo–1Co heat-resistant steel. Microstructure analysis proved that the strength of 9Cr–1.5Mo–1Co and 9Cr–3W–3Co heat-resistant steel is affected by grain boundary, dislocation, precipitation, and solid solution atoms. The excellent high temperature mechanical properties of 9Cr–3W–3Co heat-resistant steel are mainly due to the solution strengthening caused by Co and W atoms and the high-density dislocations distributed in the matrix; however, 9Cr–1.5Mo–1Co heat-resistant steel is mainly due to the martensitic lath and precipitation strengthening.

## 1. Introduction

The consumption of coal is the main source of air pollution, especially the emission of CO_2_, which leads to the increasingly serious greenhouse effect. It is a hot topic in today’s society how to reduce greenhouse gas emissions and environmental pressure [1,2]. Thermal power plant is the main enterprise of coal resource consumption. In order to reduce CO_2_ emissions under ensuring social and economic development, it is necessary to improve the efficiency of thermal power generation. To achieve this goal, it is necessary to the increase the temperature and pressure of the main steam engine, which puts forward higher requirements for materials [3,4,5,6,7]. Because of its high creep strength, good temperature oxidation resistance, excellent thermal conductivity, low thermal expansion coefficient, and low sensitivity to thermal fatigue performance, 9–12% Cr martensitic heat-resistant steel has become the heat-resistant material in thermal power plants [8,9,10].

The unique tempered martensite microstructure of 9–12% Cr heat-resistant steel is the key to keep good mechanical properties at a high temperature. The strengthening mechanisms are mainly grain boundary strengthening, precipitation strengthening, dislocation strengthening, and solution strengthening [11,12]. The grain boundary of 9–12% Cr martensitic heat-resistant steel is mainly composed of original austenite grain boundary, sub grain boundary, and martensitic lath boundary, whose properties are directly related to the mechanical properties of heat-resistant steel. In addition, the high-density dislocations in martensitic heat-resistant steel play an important role in improving the mechanical properties of heat-resistant steel [13]. Thirdly, the precipitates with different sizes can hinder the movement of dislocations and grain boundaries, such as M_23_C_6_ carbides and MX carbonitrides [14,15,16], which makes the material from high temperature tensile property and creep resistance [17,18]. Finally, large and small size solute atoms exist in the matrix, which plays a solid solution strengthening role in heat-resistant steel.

Lots of work has been done on the strengthening mechanism of 9–12% Cr martensitic heat-resistant steel. Yao Du et al. [19] found that the formation of MX precipitate is the main reason for the strengthening of 0.032C–9Cr–0.07Nb–0.13Mo–0.23Si–1.5Mn–0.8Ni heat-resistant steel. Peng Yan et al. [20,21] proved that dislocation and lath evolution are the main factors affecting the high-temperature strength of G115 heat-resistant steel, and the effect of precipitation strengthening is less than that of dislocation and lath strengthening, while the precipitated phase mainly hinders the movement of dislocation and lath boundary. At the same time, the decrease of supersaturation degree of interstitial atoms will also cause the decrease of mechanical properties of heat-resistant steel. S.S. Wang et al. [22] found that 9Cr–0.5Mo–1.8W heat-resistant steel was strengthened mainly by the combination of grain boundary strengthening, dislocation strengthening, and precipitation strengthening.

9Cr–1.5Mo–1Co and 9Cr–3W–3Co heat-resistant steel are two important members of 9–12% Cr martensitic heat-resistant steel. However, there are few studies in the literature comparing the strengthening mechanism of these two kinds of heat-resistant steels. 9Cr–1.5Mo–1Co heat-resistant steel is the heat-resistant material currently used in 620 °C thermal power generation units, while 9Cr–3W–3Co heat-resistant steel is a newly developed type of steel used in 650 °C thermal power generation units. The high temperature tensile properties of the two steels were compared and the mechanism was discussed. It is of great significance to the application and development of 9Cr–3W–3Co heat-resistant steel. At the same time, it is conducive to the development of new materials.

In this paper, 9Cr–1.5Mo–1Co and 9Cr–3W–3Co heat-resistant steels are taken as the research objects. The microstructure and high-temperature mechanical properties of 9Cr–1.5Mo–1Co and 9Cr–3W–3Co heat-resistant steels are studied comparatively, and their strengthening and toughening mechanism is revealed.

## 2. Materials and Methods 

The steel used in this study was melted in an electric furnace, refining furnace, and vacuum refining furnace; then obtained by sand casting; and finally normalized at 1000 °C and tempered at 700 °C, and the chemical composition is shown in Table 1. A sample of 10 mm × 10 mm × 10 mm is cut from the sample by wire cutting. After grinding and mechanical polishing, the sample is corroded with the etchant prepared by FeCl_3_ (5 g) + HCl (15 mL) + H_2_O (80 mL) for 20 s, and the corroded sample is washed and dried with alcohol. The microstructure and the distribution of the main elements were observed and analyzed by optical microscope (OM), scanning electron microscope (SEM), and electron probe microanalyzer (EPMA). SEM was performed on JSM-6700F (JEOL, Tokyo, Japan) with an operating voltage of 15 kV. The EPMA scan was performed on EPMA-8050G (Shimadzu, Kyoto, Japan) with an operating voltage of 15 kV. The transmission sample was prepared by the tenupol-5 electrolytic double spray instrument (Struers, Ballerup, Denmark).The double spray liquid was 4% perchloric acid ethanol solution, which is cooled to −30 °C by liquid nitrogen, and the voltage was 20 V. Precipitates, laths, and dislocations were observed on thin foils using JEM-2100 emission transmission electron microscopy (JEOL, Tokyo, Japan) at 200 kV. Thermodynamic calculation was performed by Thermo-Calc software (Thermo-Calc-2015a, TCS, Solna, Sweden) and TCFe7 database was used.

**Figure 1:**
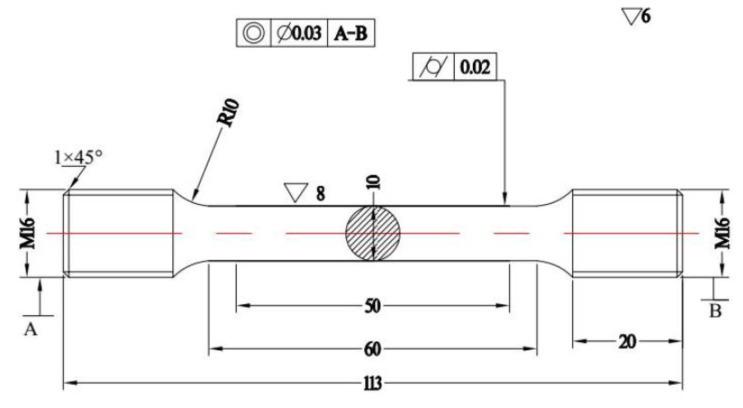
Sample size of high temperature tensile test.

Zwick Z1100TEW universal material testing machine (Zwick/Roell, Kennesaw, GA, USA) was used to measure the high temperature tensile properties. The sample size for high temperature tensile property test is shown in Figure 1 The experimental temperatures were 25 °C, 200 °C, 400 °C, 500 °C, 650 °C, and 700 °C, respectively. Three groups of experiments were carried out at each temperature to ensure the reliability of the experiment. The sample is heated to the required temperature within 60 min, which is held for 15 min at the required temperature, and then the tensile property test is started. The strain rate of the tensile test is 0.015 min^−1^. After the tensile test, scanning electron microscope (SEM) was used to observe the morphology and analyze the fracture mechanism.

## 3. Results

### 3.1. Evolution of High-Temperature Strength under As-Received

**Figure 2:**
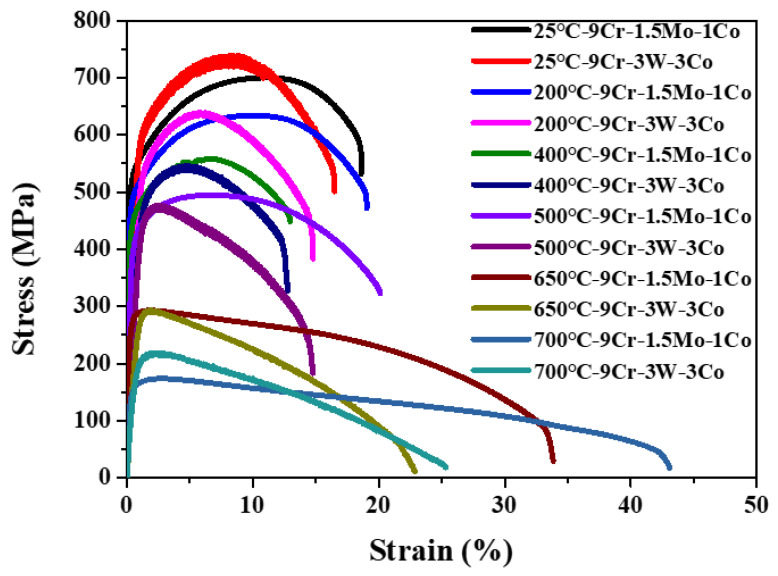
The stress–strain curves of 9Cr–1.5Mo–1Co and 9Cr–3W–3Co heat-resistant steel.

The high temperature tensile curves of 9Cr–1.5Mo–1Co and 9Cr–3W–3Co heat-resistant steel are shown in Figure 2. It can be seen from the figure that the tensile strength and yield strength of 9Cr–1.5Mo–1Co and 9Cr–3W–3Co heat-resistant steel decrease with the increase of temperature. However, elongation and reduction of area decreased or remained unchanged before 400 °C, and increased with the increase of tensile temperature after 400 °C. At a high temperature, the diffusion ability of metal atom is enhanced, the atom is easy to move, the pinning effect of defect on dislocation is weakened, the resistance of dislocation motion is reduced, and the macroscopic strength is reduced. 

**Figure 3:**
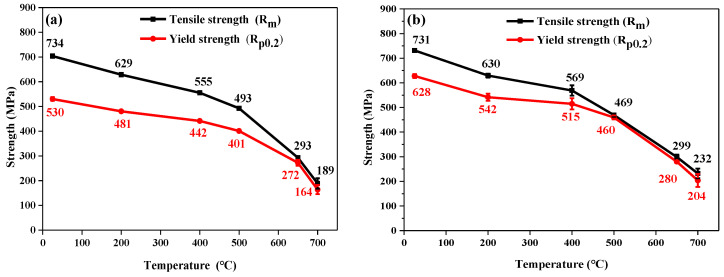
Relationship between the temperature and strength of heat resistant steel at different temperatures: (**a**) 9Cr–1.5Mo–1Co and (**b**) 9Cr–3W–3Co.

The relationship between the temperature and strength of 9Cr–1.5Mo–1Co and 9Cr–3W–3Co heat-resistant steel is shown in Figure 3. It can be seen from the figure that the overall strength of 9Cr–1.5Mo–1Co and 9Cr–3W–3Co heat-resistant steel decreases with the increase of tensile temperature. There is no obvious regularity difference between 9Cr–1.5Mo–1Co and 9Cr–3W–3Co heat-resistant steel of the tensile strength, and the difference between them is within 30MPa. However, compared with 9Cr–1.5Mo–1Co heat-resistant steel, the yield strength of 9Cr–3W–3Co heat-resistant steel is significantly higher than that of 9Cr–1.5Mo–1Co heat-resistant steel. At room temperature, the yield strength of 9Cr–1.5Mo–1Co heat-resistant steel is 530 MPa, which is about 100 MPa lower than that of 9Cr–3W–3Co heat-resistant steel. When the tensile temperature rises to 500 °C, the yield strength of 9Cr–1.5Mo–1Co heat-resistant steel is 401 MPa, which is about 60MPa lower than that of 9Cr–3W–3Co heat-resistant steel. When the tensile temperature rises to 700 °C, the yield strength of 9Cr–1.5Mo–1Co heat-resistant steel is 164 MPa, which is about 40MPa lower than that of 9Cr–3W–3Co heat-resistant steel. 

**Figure 4:**
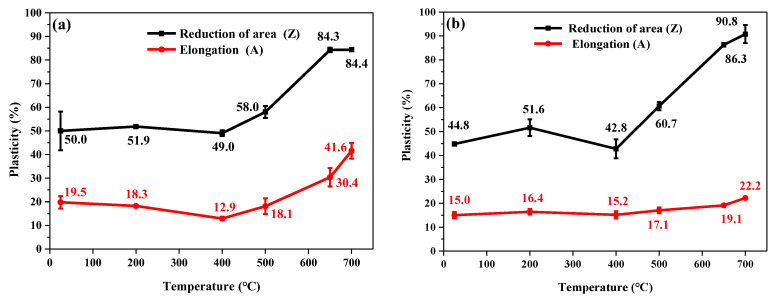
Relationship between temperature and plasticity of heat-resistant steel at different temperatures: (**a**) 9Cr–1.5Mo–1Co and (**b**) 9Cr–3W–3Co.

The relationship between the temperature and plasticity of 9Cr–1.5Mo–1Co and 9Cr–3W–3Co heat-resistant steel is shown in Figure 4. It can be seen from the figure that the plastic change rule of 9Cr–1.5Mo–1Co and 9Cr–3W–3Co heat-resistant steel is similar. When the tensile temperature is less than 400 °C, the elongation and area shrinkage of 9Cr–1.5Mo–1Co and 9Cr–3W–3Co heat-resistant steel do not change significantly with the rise of temperature. When the tensile temperature is greater than 400 °C, the elongation and reduction of area of 9Cr–1.5Mo–1Co and 9Cr–3W–3Co heat-resistant steel began to rise sharply. However, the overall elongation of 9Cr–3W–3Co heat-resistant steel is lower than that of 9Cr–1.5Mo–1Co heat-resistant steel, and there is no significant difference between the 9Cr–1.5Mo–1Co and 9Cr–3W–3Co heat-resistant steel. At room temperature, the elongation of 9Cr–1.5Mo–1Co heat-resistant steel is 19.5% and the reduction of area is 50.0%, however, the elongation and reduction of area of 9Cr–3W–3Co heat-resistant steel are 15% and 44.8% respectively, that is, the elongation and area reduction of 9Cr–1.5Mo–1Co heat-resistant steel are 4.5% and 5.2% higher than those of 9Cr–3W–3Co heat-resistant steel, respectively. When the tensile temperature is increased to 650 °C, the elongation of 9Cr–1.5Mo–1Co heat-resistant steel is increased by 30.4% and the reduction of area is increased by 58.0%, but the elongation and area reduction of 9Cr–3W–3Co heat-resistant steel are 19.1% and 86.3% respectively. In other words, the elongation and area reduction of 9Cr–1.5Mo–1Co heat-resistant steel are 11.3% higher and 2.0% lower those of 9Cr–3W–3Co heat-resistant steel, respectively. Compared with plasticity, strength is the most important index to evaluate the properties of heat-resistant steel. Under the service temperature of heat-resistant steel, the atomic diffusion speed is faster, and the plasticity of materials will rise to a certain extent. Therefore, plasticity is generally not the focus of heat-resistant steel research.

### 3.2. Analysis of Fracture Morphology after High Temperature Tensile

**Figure 5:**
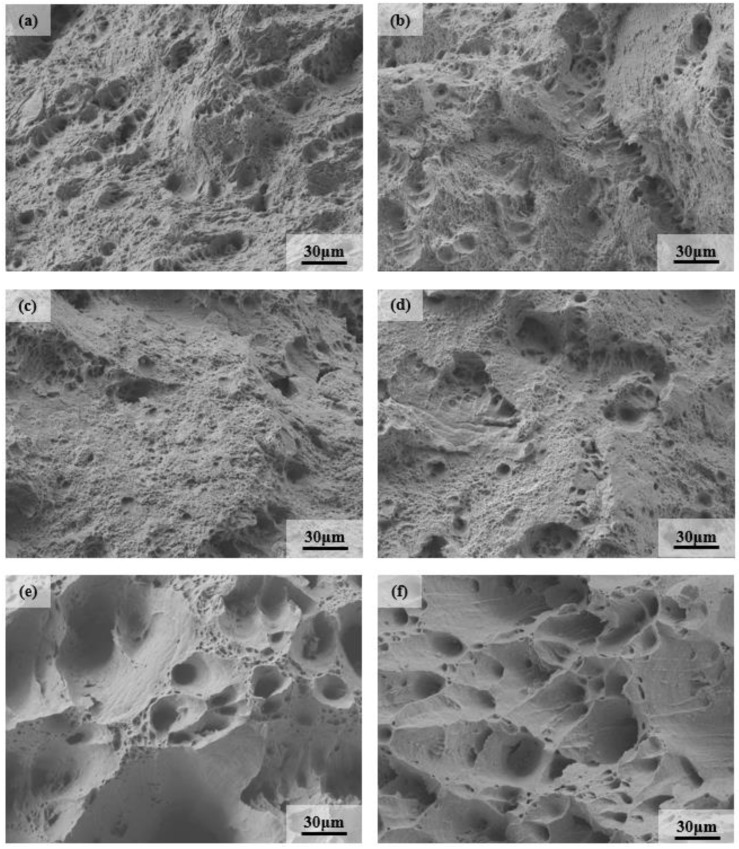
Tensile fracture of 9Cr–1.5Mo–1Co heat resistant steel at different temperatures: (**a**) 25 °C, (**b**) 200 °C, (**c**) 400 °C, (**d**) 500 °C, (**e**) 650 °C, and (**f**) 700 °C.

**Figure 6:**
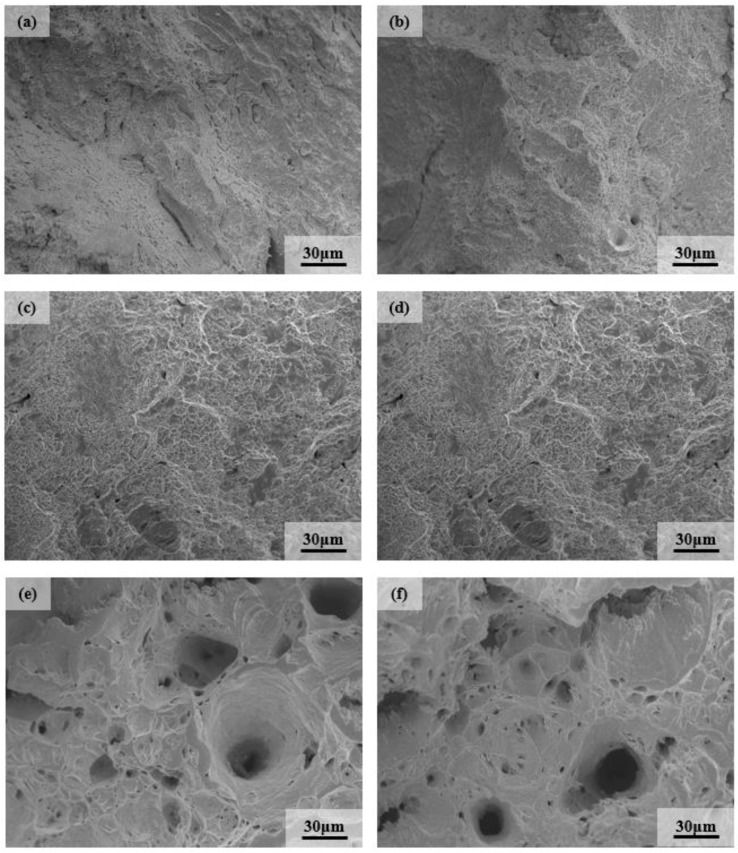
Tensile fracture of 9Cr–3W–3Co heat resistant steel at different temperatures: (**a**) 25 °C, (**b**) 200 °C, (**c**) 400 °C, (**d**) 500 °C, (**e**) 650 °C, and (**f**) 700 °C.

The tensile fracture of 9Cr–1.5Mo–1Co and 9Cr–3W–3Co heat-resistant steel at different temperatures is shown in Figure 5 and Figure 6. It can be seen from the figure that 9Cr–1.5Mo–1Co and 9Cr–3W–3Co heat-resistant steel are mainly ductile fracture. With the increase of temperature, the dimple deepens and the size increases, especially at 650 °C, the dimple on the fracture surface becomes larger and deeper. Because there are many second phase particles in 9Cr–1.5Mo–1Co and 9Cr–3W–3Co that are heat-resistant, these second phase particles can effectively resist crack growth at a high temperature, which also shows that plasticity increases with the increase of tensile temperature. This is also confirmed by the results of elongation and reduction of area, that is to say, the plasticity of the material above 650 °C is obviously improved.

### 3.3. Microstructural Characterization of the As-Received Steel

**Figure 7:**
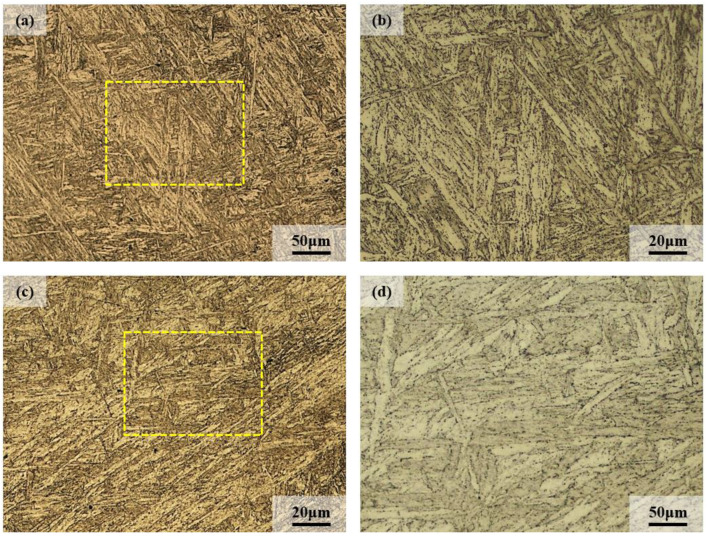
Optical microscope (OM) image of heat-resistant steel after heat treatment: (**a**,**b**) 9Cr–1.5Mo–1Co, (**c**,**d**) 9Cr–3W–3Co.

The OM images of 9Cr–1.5Mo–1Co and 9Cr–3W–3Co heat-resistant steel are shown in Figure 7. It can be seen from the figure that the microstructures of 9Cr–1.5Mo–1Co and 9Cr–3W–3Co heat-resistant steel are typical tempered martensite. After high-temperature tempering, the martensite morphology is relatively complete, and some martensite laths are relatively wide. It is possible that during tempering, the martensite lath has merged, and at the same time, there are a large number of black dot like precipitates on the edge of the martensite lath.

**Figure 8:**
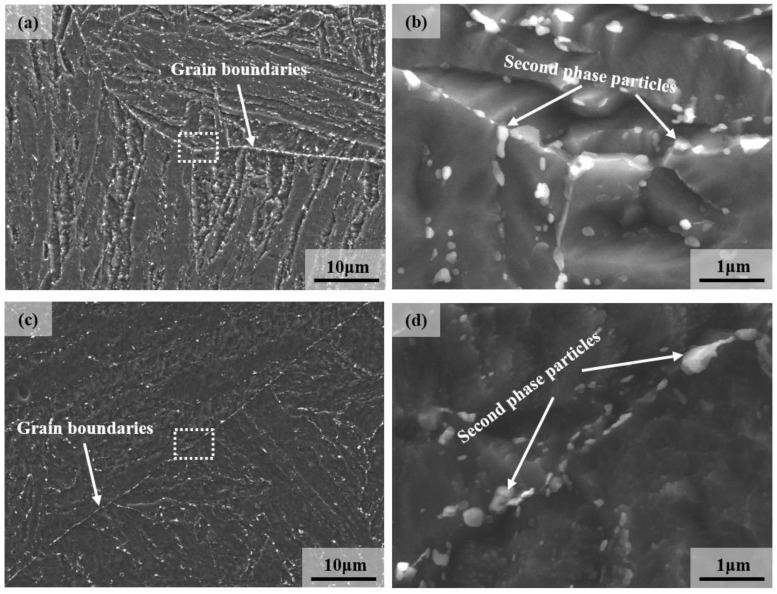
Scanning electron microscope (SEM) image of heat-resistant steel after heat treatment: (**a**,**b**) 9Cr–1.5Mo–1Co, (**c**,**d**) 9Cr–3W–3Co.

SEM images of 9Cr–1.5Mo–1Co and 9Cr–3W–3Co heat-resistant steel are shown in Figure 8. It can be seen from the figure that there are precipitates with different sizes in the grain boundary, the edge of martensitic lath, and the interior of martensitic lath, and compared with the precipitates at the edge of martensitic lath and the interior of martensitic lath, the precipitates at the grain boundary have larger sizes and obvious clustering phenomenon. The obvious difference is mainly due to the higher energy at the grain boundary, which is conducive to the precipitation phase nucleation and carbide aggregation. At the same time, it can be found that the number of precipitates in 9Cr–1.5Mo–1Co heat-resistant steel is more than that in 9Cr–3W–3Co heat-resistant steel with the same area. According to the statistics of software IPP, the average size of precipitates in 9Cr–1.5Mo–1Co heat-resistant steel is 156.5 nm, and the average size of precipitates in 9Cr–3W–3Co heat-resistant steel is 270.7 nm.

**Figure 9:**
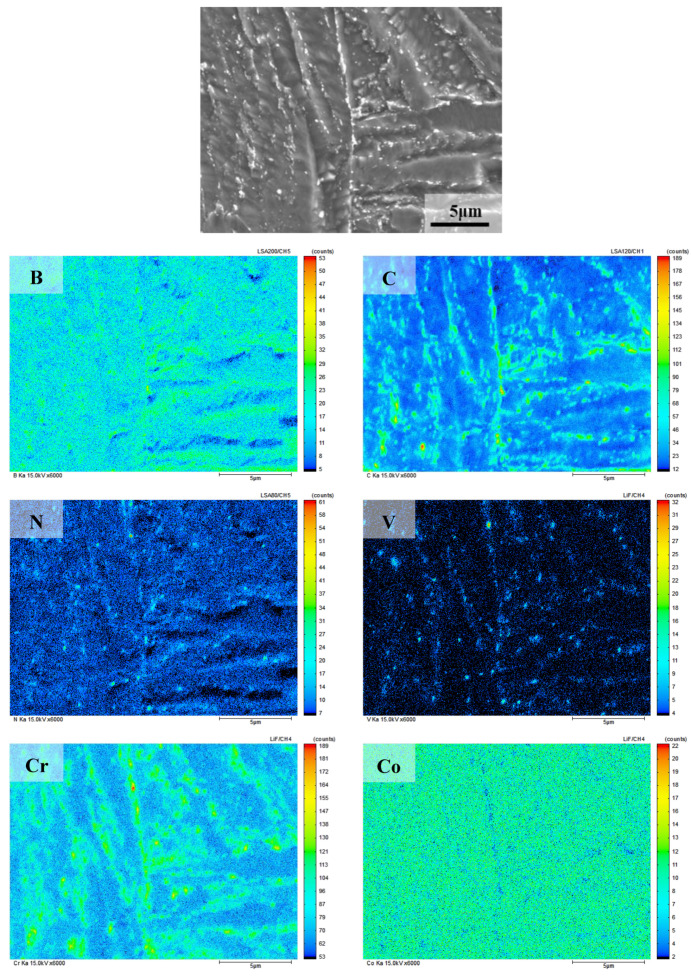

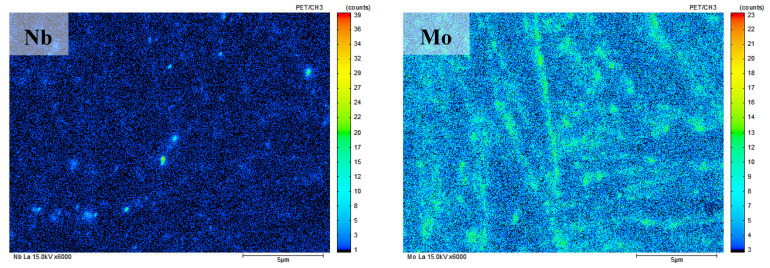
Analysis results of elements in 9Cr–1.5Mo–1Co heat-resistant steel.

The results of surface element in electron probe microanalyzer(EPMA)of 9Cr–1.5Mo–1Co heat-resistant steel are shown in Figure 9. It can be seen from the figure that C, N, V, Cr, Nb, Mo, and other elements are in the state of segregation. According to the relevant literature [23,24], a large number of carbide in the grain boundary and phase boundary may be M_23_C_6_, while MX precipitate may be formed inside the martensitic lath, and Co element does not participate in the formation of precipitate, which is evenly distributed in the matrix.

**Figure 10:**
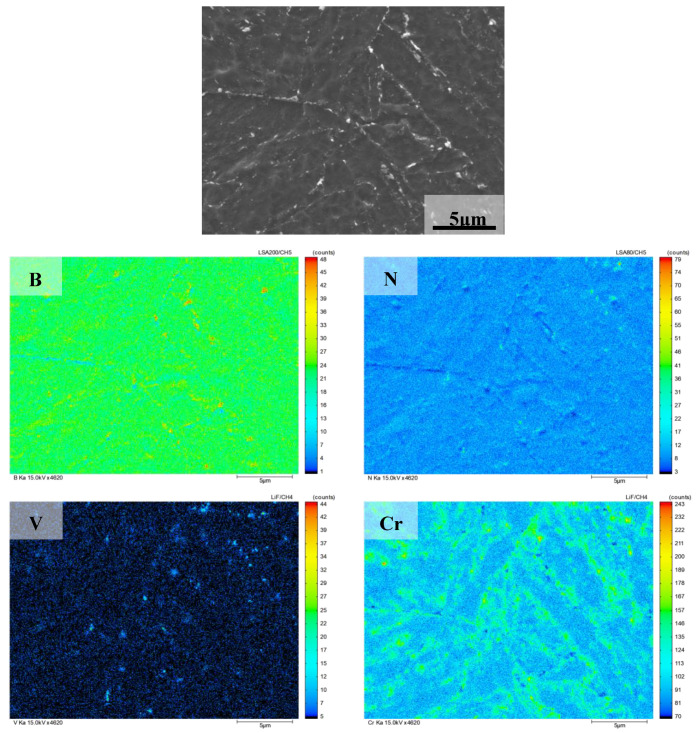

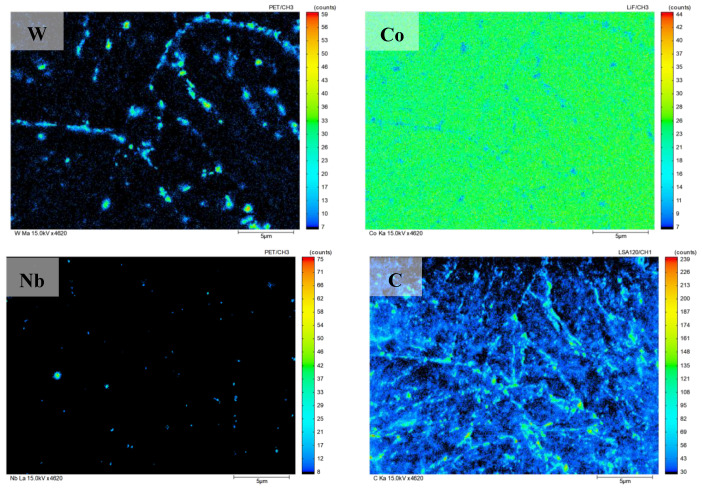
Analysis results of elements in 9Cr–3W–3Co heat-resistant steel.

The results of surface element in EPMA of 9Cr–3W–3Co heat-resistant steel are shown in Figure 10. Similar to 9Cr–1.5Mo–1Co, it can be seen from the figure that C, N, V, Cr, Nb, W, and other elements are in the state of segregation. According to the relevant literature [23,24], a large number of carbide in the grain boundary and phase boundary may be M_23_C_6_, while MX precipitate may be formed inside the martensitic lath, and Co element does not participate in the formation of precipitate, which is evenly distributed in the matrix.

**Figure 11:**
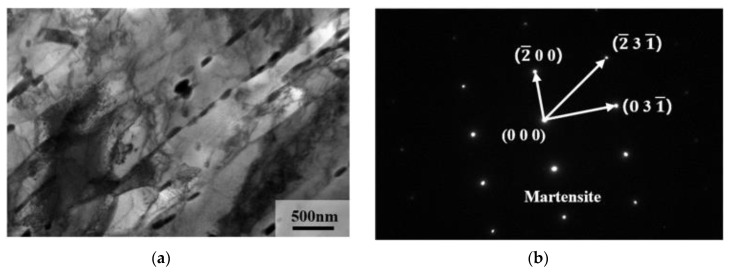

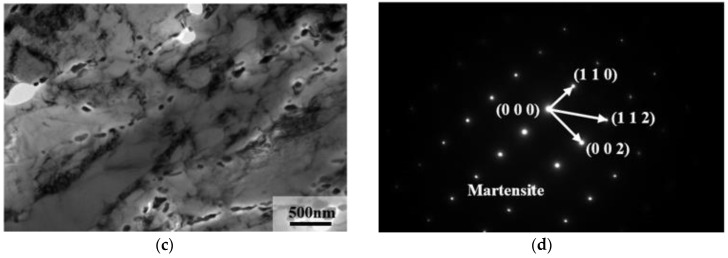
Transmission electron microscopy (TEM) image of heat-resistant steel after heat treatment: (**a**,**b**) 9Cr–1.5Mo–1Co, (**c**,**d**) 9Cr–3W–3Co.

TEM images of the matrix microstructure of 9Cr–1.5Mo–1Co and 9Cr–3W–3Co heat-resistant steel are shown in Figure 11. It can be seen from the figure that 9Cr–1.5Mo–1Co and 9Cr–3W–3Co heat-resistant steel are both typical lath-like structures with obvious precipitation at the lath boundary. Through the analysis of crystal structure, 9Cr–1.5Mo–1Co and 9Cr–3W–3Co heat-resistant steels are martensitic microstructure. At the same time, it is found that the width of the martensitic lath of 9Cr–1.5Mo–1Co heat-resistant steel is significantly smaller than that of 9Cr–3W–3Co heat-resistant steel. The width of the martensitic lath of 9Cr–1.5Mo–1Co heat-resistant steel is 385 ± 55 nm, but that of 9Cr–3W–3Co heat-resistant steel is 495 ± 55 nm.

**Figure 12:**
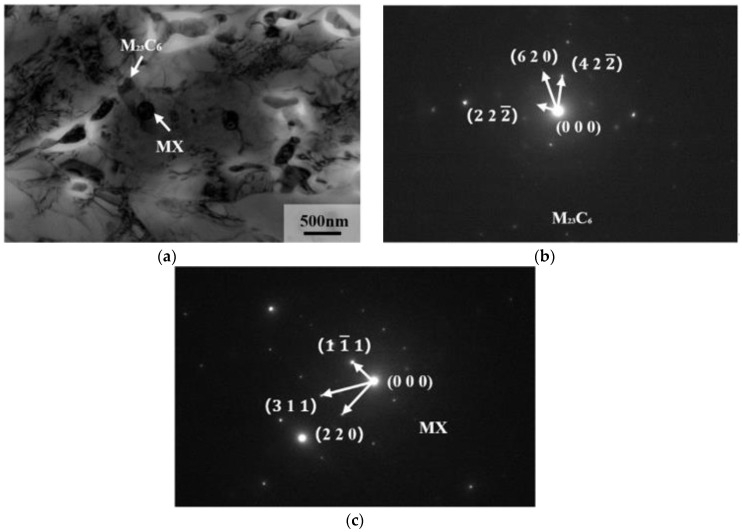
TEM image of precipitation phase in 9Cr–1.5Mo–1Co heat-resistant steel: (**a**) the morphology of precipitated phase, (**b**) diffraction pattern of M_23_C_6_ precipitates, (**c**) diffraction pattern of MX precipitates.

**Figure 13:**
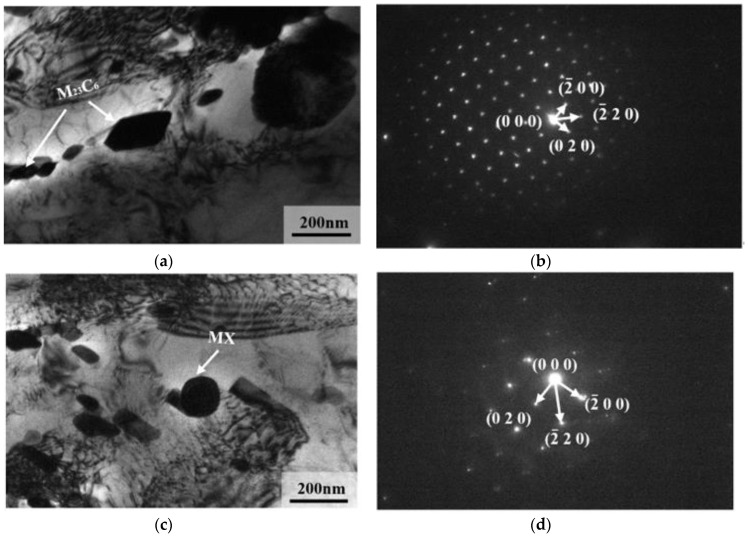
TEM image of precipitation phase in 9Cr–3W–3Co heat-resistant steel: (**a**) the morphology of M_23_C_6_ precipitates, (**b**) diffraction pattern of M_23_C_6_ precipitates, (**c**) the morphology of MX precipitates, (**d**) diffraction pattern of MX precipitates

**Figure 14:**
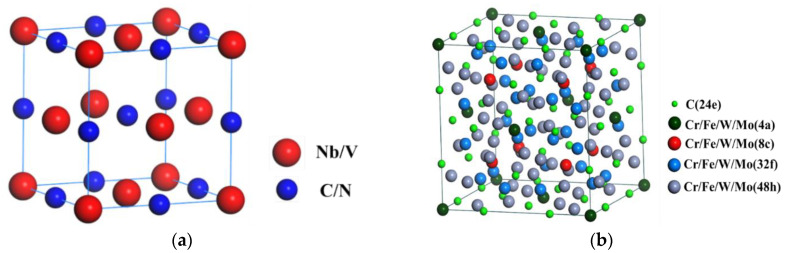
Structure diagram of M_23_C_6_ precipitate and MX precipitates: (**a**) crystal structure of MX precipitate, (**b**) crystal structure of M_23_C_6_ precipitate.

TEM images of precipitated phases in 9Cr–1.5Mo–1Co and 9Cr–3W–3Co heat-resistant steel are shown in Figure 12 and Figure 13. It can be seen from the figure that the precipitates in 9Cr–1.5Mo–1Co and 9Cr–3W–3Co heat-resistant steel are M_23_C_6_ precipitates and MX precipitates. The structure of these two precipitates is shown in Figure 14. For M_23_C_6_ precipitates, the metal atoms are distributed in 4a, 8c, 32f, and 48h of the cell, while the C atoms are mainly located in 24e. In the MX precipitate, the metal atom is located at the top of the cell and the center of the crystal surface, while the non-metal atom is located at the center of the edge and the center of the crystal cell. Because of the different positions of formation, M_23_C_6_ precipitates are formed at the lath boundary and sub grain boundary, MX precipitates are formed inside the martensitic lath, M_23_C_6_ precipitates are elliptical, and MX precipitates are spherical.

**Figure 15:**
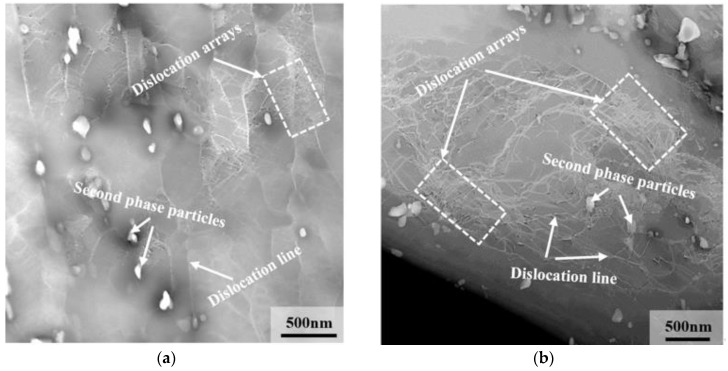
Dislocation distribution of heat-resistant steel under TEM after heat treatment: (**a**) 9Cr–1.5Mo–1C0 and (**b**) 9Cr–3W–3Co.

The dislocation distribution in 9Cr–1.5Mo–1Co and 9Cr–3W–3Co heat-resistant steel is shown in Figure 15. It can be seen from the figure that a large number of simple dislocation modes, such as dislocation line and dislocation accumulation, are distributed in the martensitic lath of 9Cr–1.5Mo–1Co and 9Cr–3W–3Co heat-resistant steel, as shown by the white arrow in the figure. The existence of these dislocation structures will strengthen the heat-resistant steel [25,26,27]. At the same time, some dislocations are distributed around the precipitates, which will further strengthen the material. When the material is deformed as a result of the force, the dislocations will move correspondingly, but the dislocations cannot cut through these hard precipitates. In other words, the hard precipitates will hinder the wrong movement, so that the matrix can be strengthened [28]. At the same time, it is easy to see the dislocation distribution in 9Cr–1.5Mo–1Co and 9Cr–3W–3Co heat-resistant steel. The dislocations in 9Cr–3W–3Co heat-resistant steel are more concentrated, and the distribution and the number of dislocation lines around the precipitated phase are obviously more than that in 9Cr–1.5Mo–1Co heat-resistant steel.

## 4. Discussion

Through the above results, it is not difficult to find that the overall mechanical properties of 9Cr–3W–3Co heat-resistant steel are significantly better than those of 9Cr–1.5Mo–1Co heat-resistant steel, which provides the possibility for 9Cr–3W–3Co heat-resistant steel to be widely used in 650 °C thermal power generating units. This part is mainly used to explain the reason that the properties of 9Cr–3W–3Co heat-resistant steel are better than those of 9Cr–1.5Mo–1Co heat-resistant steel.

9Cr–1.5Mo–1Co and 9Cr–3W–3Co heat-resistant steel belong to 9–12% Cr martensitic heat-resistant steel. Chromium equivalent directly affects the overall performance of heat-resistant steel. The chromium equivalent is calculated as follows [29]:

NCE = %Cr + 6 × %Si + 4 × %Mo+1.5 × %W + 11 × %V + 5 × %Nb + 12 × %Al + 8 × %Ti − 40 × %C − 2 × %Mn − 4 × %Ni − 2 × %Co − 30 × %N − %Cu(1)

**Figure 16:**
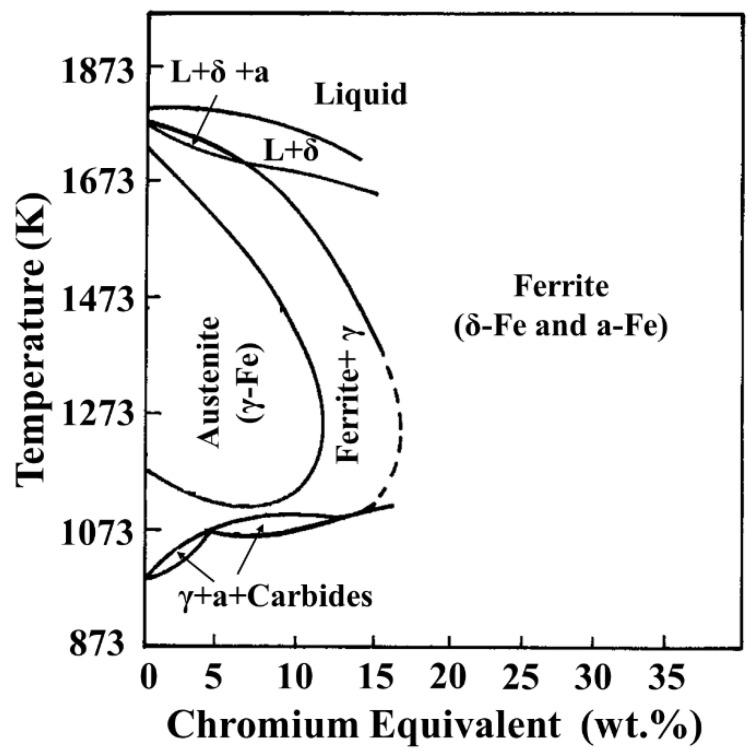
Pseudoequilibrium diagram showing the dependence of the existence of various phases on the chromium equivalent.

After calculation, the chromium equivalent of 9Cr–1.5Mo–1Co heat-resistant steel is 8.992, while that of 9Cr–3W–3Co heat-resistant steel is 7.783. Compared with Figure 16, it is not difficult to find that 9Cr–1.5Mo–1Co and 9Cr–3W–3Co heat-resistant steels are in the austenite phase area when they are heat treated in the same austenite, which ensures that δ–Fe transformation does not occur in the process of austenitizing, that is to say, the microstructure of 9Cr–1.5Mo–1Co and 9Cr–3W–3Co heat-resistant steel is the cause of tempered martensite. However, according to the analysis of martensitic structure of 9Cr–1.5Mo–1Co and 9Cr–3W–3Co heat-resistant steel in Figure 11, the width of the martensitic lath of 9Cr–1.5Mo–1Co heat-resistant steel is obviously smaller than that of 9Cr–3W–3Co heat-resistant steel, and the smaller the martensitic lath, the better the mechanical properties. Therefore, the influence of matrix structure on the strength of 9Cr–1.5Mo–1Co heat-resistant steel is greater than that on 9Cr–3W–3Co heat-resistant steel.

In addition to grain boundary strengthening, the strengthening methods of 9–12% Cr martensitic heat-resistant steel mainly include solution strengthening, precipitation strengthening, and dislocation strengthening. It can be seen from the above experimental results that the microstructures of 9Cr–1.5Mo–1Co and 9Cr–3W–3Co heat-resistant steel are similar, and there are four strengthening ways mentioned above. Therefore, it is necessary to distinguish the effect of the other three strengthening mechanisms on the two heat-resistant steels.

**Figure 17:**
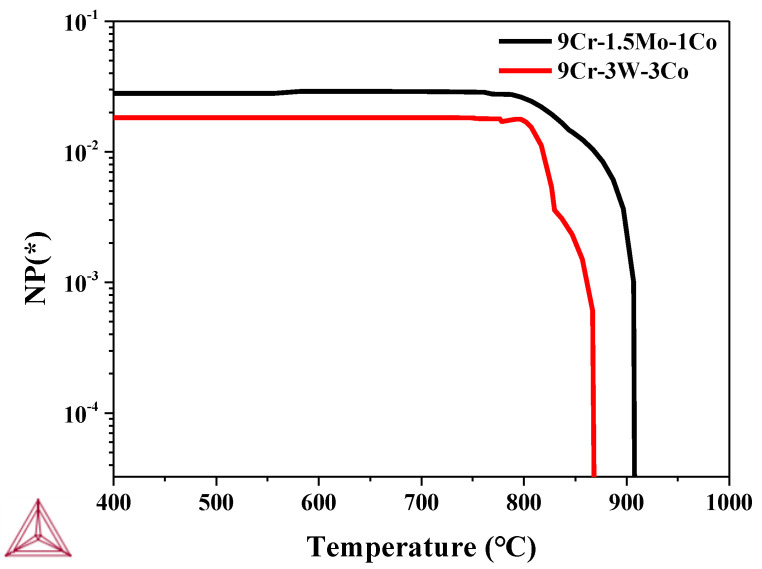
Change of precipitation amount of M_23_C_6_ precipitate with temperature.

The pinning force PZ of the precipitated relative grain boundary or sub grain boundary and the pinning force PB for the martensitic lath can be expressed as follows [15]:

(2) $$P_{Z}=\frac{3\gamma F_{V}}{d}$$

(3) $$P_{B}=\frac{\gamma F_{VB}D}{d^{2}}$$

where *γ* is the boundary surface energy per unit area, *F_V_* and *F_VB_* are volume fraction, and *d* is the size of dispersed particles, respectively. *D* represents the size of structural elements, that is, the grain/subgrain size or lath thickness. It can be seen from the formula that the pinning force of precipitation relative to grain boundary or martensitic lath boundary is mainly related to the volume fraction and the size of precipitated phase. With the increase of the volume fraction of precipitates, the pinning effect of precipitates is stronger, that is, it is more favorable for the strength of heat-resistant steel. With the increase of the size of precipitates, the pinning effect of precipitates is weaker, that is, it is more unfavorable for the strength of heat-resistant steel. Figure 17 shows the precipitation amount of M_23_C_6_ in 9Cr–1.5Mo–1Co and 9Cr–3W–3Co heat-resistant steel calculated by Thermo-Calc software. It can be seen from the figure that the precipitation of M_23_C_6_ in 9Cr–1.5Mo–1Co heat-resistant steel is significantly greater than that of M_23_C_6_ in 9Cr–3W–3Co heat-resistant steel, which is mutually confirmed with the experimental results in Figure 8. According to the statistical results of precipitate size in Figure 8, the average size of precipitate in 9Cr–1.5Mo–1Co heat-resistant steel is smaller than that in 9Cr–3W–3Co heat-resistant steel. In conclusion, the pinning force of 9Cr–3W–3Co heat-resistant steel is smaller than that of 9Cr–1.5Mo–1Co heat-resistant steel.

The strengthening effect of dislocation strengthening on steel can be calculated by the following formula [30]:

(4) $$\sigma_{\rho }=0.5{MGb\rho }^{1/2}$$

where *M* is the Taylor coefficient, *G* is the shear modulus, *b* is the length of Burgers vector, and ρ is the dislocation density. It can be seen that the strength of the material increases with the increase of dislocation density. According to the analysis results in Figure 15, the dislocation density of 9Cr–3W–3Co heat-resistant steel is significantly higher than that of 9Cr–1.5Mo–1Co heat-resistant steel. Therefore, the contribution of dislocation to the strength of 9Cr–3W–3Co heat-resistant steel is higher than that of 9Cr–1.5Mo–1Co heat-resistant steel.

Solution strengthening is mainly related to the radius difference between the solute atom and matrix atom. The larger the radius difference between the solute atom and matrix atom, the stronger the strengthening effect on the matrix. Therefore, in 9Cr–1.5Mo–1Co and 9Cr–3W–3Co heat-resistant steels, except for the small-size non-metallic elements such as C, N, and B, which are strengthened in the form of interstitial atoms, the metal elements such as Co, W, and Mo can also strengthen 9Cr–1.5Mo–1Co or 9Cr–3W–3Co heat-resistant steel in the form of replacement atoms. According to Figure 9 and Figure 10, Co atoms mainly exist in the matrix, while some Mo and W atoms are precipitated in the form of precipitated phase, and the rest are also solid soluble in the matrix. Combined with the element composition of 9Cr–3W–3Co or 9Cr–1.5Mo–1Co, it can be seen that the Co element content of 9Cr–3W–3Co is higher than that of 9Cr–1.5Mo–1Co. Therefore, the solid solution strengthening effect of Co on 9Cr–3W–3Co is stronger than that of 9Cr–1.5Mo–1Co. In addition, W element and Mo element belong to the same group, and the atomic number of W atom is greater than that of Mo atom, and the effect of W atom on steel solution strengthening is greater than that of Mo atom on steel. In conclusion, the strengthening effect of Co and W atoms on 9Cr–3W–3Co heat-resistant steel is higher than that of Co and Mo atoms on 9Cr–1.5Mo–1Co heat-resistant steel.

## 5. Conclusions

On the basis of the analysis of the tensile properties and strengthening mechanism of 9Cr–1.5Mo–1Co and 9Cr–3W–3Co heat-resistant steel at different temperatures, the following conclusions are obtained:

1. At the same temperature, the overall yield strength of 9Cr–3W–3Co heat-resistant steel is better than that of 9Cr–1.5Mo–1Co heat-resistant steel. At room temperature, the yield strength of 9Cr–3W–3Co heat-resistant steel is 628 MPa, while that of 9Cr–1.5Mo–1Co heat-resistant steel is 530 MPa. When the tensile temperature is increased to 700 °C, the yield strength of 9Cr–3W–3Co heat-resistant steel is 204 MPa, while that of 9Cr–1.5Mo–1Co heat-resistant steel is 164 MPa.
2. The strength of 9Cr–3W–3Co heat-resistant steel and 9Cr–1.5Mo–1Co heat-resistant steel is affected by grain boundary, dislocation, precipitation, and solid solution atoms, but the width of the martensitic lath of 9Cr–1.5Mo–1Co heat-resistant steel is smaller than that of 9Cr–3W–3Co heat-resistant steel, and the dislocation density of 9Cr–3W–3Co heat-resistant steel is larger than that of 9Cr–1.5Mo–1Co heat-resistant steel.
3. The excellent high-temperature mechanical properties of 9Cr–3W–3Co heat-resistant steel are mainly attributed to the solution strengthening caused by Co and W atoms and the high-density dislocations distributed in the matrix, while the precipitates are mainly pinned dislocations and hinder the movement of dislocations. However, the high temperature strength of 9Cr–1.5Mo–1Co heat-resistant steel is mainly due to the refinement and precipitation of the martensitic lath and the pinning of the grain boundary.

## Figures and Tables

**Table 1:** Chemical composition of the steel, wt%.

Element	C	Si	Mn	Cr	Mo	Ni	V	Nb	Co	W
9Cr-1.5Mo-1Co	0.05~0.15	0.2~0.4	0.5~1	9~12	1.5	0~0.3	0~0.3	0~0.1	1.00	0
9Cr-3W-3Co	0.05~0.15	0.2~0.4	0.5~1	9~12	0	0~0.3	0~0.3	0~0.1	3.00	2.80
